# Sex differences in stress-related receptors: ″micro″ differences with ″macro″ implications for mood and anxiety disorders

**DOI:** 10.1186/2042-6410-4-2

**Published:** 2013-01-21

**Authors:** Debra A Bangasser

**Affiliations:** 1 Department of Psychology and Neuroscience Program, Temple University, 1701 North 13th Street, 873 Weiss Hall, Philadelphia, 19122, PA

**Keywords:** Corticotropin releasing factor, Glucocorticoids, Depression, Anxiety disorder, Gender difference

## Abstract

Stress-related psychiatric disorders, such as unipolar depression and post-traumatic stress disorder (PTSD), occur more frequently in women than in men. Emerging research suggests that sex differences in receptors for the stress hormones, corticotropin releasing factor (CRF) and glucocorticoids, contribute to this disparity. For example, sex differences in CRF receptor binding in the amygdala of rats may predispose females to greater anxiety following stressful events. Additionally, sex differences in CRF receptor signaling and trafficking in the locus coeruleus arousal center combine to make females more sensitive to low levels of CRF, and less adaptable to high levels. These receptor differences in females could lead to hyperarousal, a dysregulated state associated with symptoms of depression and PTSD. Similar to the sex differences observed in CRF receptors, sex differences in glucocorticoid receptor (GR) function also appear to make females more susceptible to dysregulation after a stressful event. Following hypothalamic pituitary adrenal axis activation, GRs are critical to the negative feedback process that inhibits additional glucocorticoid release. Compared to males, female rats have fewer GRs and impaired GR translocation following chronic adolescent stress, effects linked to slower glucocorticoid negative feedback. Thus, under conditions of chronic stress, attenuated negative feedback in females would result in hypercortisolemia, an endocrine state thought to cause depression. Together, these studies suggest that sex differences in stress-related receptors shift females more easily into a dysregulated state of stress reactivity, linked to the development of mood and anxiety disorders. The implications of these receptor sex differences for the development of novel pharmacotherapies are also discussed.

## Introduction

Many mood and anxiety disorders are considered stress-related, because of the crucial role stress plays in their etiology and symptomology. For example, stress is associated with the onset and severity of unipolar depression, panic disorder, and generalized anxiety disorder [1-7]. In the case of post-traumatic stress disorder (PTSD), the disease is, by definition, precipitated by a stressful event [4,8]. Additionally, early life stress can sensitize later responses to stress and is associated with the development of mood and anxiety disorders in adulthood [9-11].

A common feature shared by most stress-related psychiatric disorders is that they occur more frequently in women than in men. Women are, in fact, twice as likely as men to suffer from unipolar depression and several anxiety disorders, including PTSD [1,12-15]. This disparity is often attributed to gender differences in psychological factors, including affect, cognitive style, and social role identification [16-23]. Some commonly presumed causes for the gender gap, such as differences in the types of trauma experienced, preexisting psychiatric disorders, and sex differences in reporting, are unlikely to play a significant role because the male–female disparity remains, even after these variables have been controlled for [4,14,24,25]. Nonetheless, it is likely that some psychological factors contribute to the sex bias in stress-related disease. New research investigating molecular sex differences in stress response systems, on the other hand, indicates that *biological* factors also contribute to sex differences in the prevalence of stress-related psychiatric disorders. This review will discuss emerging data that has identified sex differences in receptors for the stress hormones, corticotropin releasing factor (CRF) and glucocorticoids. The reviewed studies suggest that receptor sex differences can cause stress responses in females to shift more easily into a dysregulated state and likely play an important role in the disparity seen between males and females in mood and anxiety rates.

## Review

### Endocrine and central effects of CRF

CRF is best known for its role in activating the hypothalamic pituitary adrenal (HPA) axis. This endocrine response is initiated during a stressful event when CRF released from neurons in the paraventricular nucleus of the hypothalamus stimulates pituitary corticotropes to secrete adrenocorticotropic hormone (ACTH) [26]. ACTH released into the bloodstream acts on the cortex of the adrenal glands causing them to release glucocorticoids (cortisol in humans and corticosterone in rodents). Glucocorticoids then feedback at the levels of the hypothalamus and pituitary to terminate the HPA axis activation [27]. Additionally, these hormones act on the hippocampus, which in turn provides negative feedback to the hypothalamus in order to aid in the termination of stress-induced activation of the HPA axis [28,29].

High levels of CRF immunoreactivity also are observed outside of the hypothalamus in other forebrain regions, such as the olfactory bulb, cerebral cortex, nucleus accumbens, septum, bed nucleus of the stria terminalis (BNST), preoptic nuclei, amygdala, and hippocampus [30-32]. Mid- and hindbrain regions that contain CRF include the periaqueductal gray, raphe nuclei, lateral tegmental nucleus, locus coeruleus (LC), parabrachial nucleus, cerebellum, and the nucleus of the solitary tract [30-32]. This widespread extra-hypothalamic expression means that CRF is in a position to centrally modulate a variety of cognitive and behavioral responses to stress. For example, CRF is known to activate the amygdala, BNST, and prefrontal cortex to mediate a number of behaviors, including those related to anxiety, defensive responses, and fear conditioning [31,33-39]. Additionally, monoaminergic neuronal transmission is regulated by CRF [40-43], further underscoring the pivotal role of CRF in coordinating a broad range of responses to stressful events.

### CRF receptor distribution and function

CRF exerts its actions via two G-protein coupled receptors: CRF_1_ receptor type (CRF_1_) and CRF_2_ receptor type (CRF_2_) [44-46]. Both CRF receptors are found in regions involved in regulating stress, mood, and anxiety. Specifically, CRF_1_ is predominantly found in the cerebral cortex, cerebellum, pons, and anterior lobe of the pituitary, while CRF_2_ is more prominent in the septum, choroid plexus, and posterior lobe of the pituitary [47-49]. In many brain regions, like the hypothalamus, hippocampus, amygdala, and BNST, there is considerable variation between the expression of CRF_1_ and CRF_2_ (e.g., CRF_1_ expression is greater in the periventricular zone of the hypothalamus, while CRF_2_ expression is greater in the ventromedial nucleus of the hypothalamus) [47-49]. This heterogeneous distribution of CRF receptor subtypes suggests that each subtype has a unique role in mediating CRF function. Genetic and pharmacological manipulations support this idea. Activation of CRF_1_ initiates the endocrine stress response and mediates anxiety-related behavior, while CRF_1_ antagonism or receptor knockout reduces anxiety [50-57]. The role of CRF_2_ in mediating stress-related behavior is more complex. Data from CRF_2_ knockout mice suggest that activation CRF_2_ attenuates activity of the HPA axis, thereby counteracting the stimulating effect of CRF_1_[58-60]. The role of CRF_2_ in mediating anxiety is less consistent with some, but not all, studies implicating these receptors in the reduction of anxiety-related behavior [50,59,61-64]. These inconsistencies may arise from the fact that some effects of CRF_2_ are region specific [65-67]. Nevertheless, the sometimes opposing actions of CRF_1_ and CRF_2_ have led to proposal that generally CRF_1_ increases while CRF_2_ decreases stress responsivity [50].

Given the widespread distribution of CRF_1_ and CRF_2_, it is clear that CRF is critically poised to modulate a number of brain regions. Thus, it is not surprising that dysregulation of CRF and its receptors can result in a number of adverse consequences. CRF hypersecretion is linked to the pathophysiology of depression and certain anxiety disorders [68-70]. High levels of CRF are found in the cerebral spinal fluid and brain regions of patients with depression and PTSD, and these levels are reduced with antidepressant treatment [70-83]. Multiple genomic studies have identified single nucleotide polymorphisms on the CRF_1_ gene that are linked to depression, panic disorder, and PTSD [84-89]. Further evidence for CRF_1_ in mediating depression comes from a clinical trial in which CRF_1_ antagonist treatment of depressed patients reduced depressed mood and anxiety symptoms [90]. Taken together, these studies suggest a causal role for CRF dysregulation in the development of stress-related pathology.

### Sex differences in CRF receptors: Evidence from knockout mice and binding studies

The psychiatric disorders linked to CRF dysregulation occur more frequently in women. Thus, it is likely that sex differences in the CRF system underlie this disparity. Indeed, sex differences in CRF expression have been observed in the paraventricular nucleus of the hypothalamus, amygdala, and BNST, with females typically having greater CRF expression [91-94]. In addition to sex differences in the expression of CRF, sex difference in CRF receptors could contribute to sex differences in stress vulnerability. Evidence for sex differences in CRF receptors comes, in part, from CRF_2_ knockout mice that have a phenotype attributable to unimpeded CRF_1_ activity [59,95]. Female CRF_2_-deficint mice display more depressive-like behaviors on the forced swim test than their male counterparts, an effect blocked by a CRF_1_ receptor antagonist [95]. Not only does this finding reveal that depressive-like behavior is mediated, at least in part, by CRF_1_, but this result suggests that sex differences at the level of the CRF_1_ contribute to increased depressive-like behavior in females. Despite this evidence for sex differences in CRF_1_, few studies have directly evaluated sex differences in CRF receptor expression and function.

Recently, Weathington and Cooke (2012) identified sex differences in CRF receptor binding in the amygdala. CRF_1_ binding was significantly greater in the basolateral and posteroventral nuclei of the amygdala in female than male rats. Interestingly, this sex difference was not identified until after puberty. Sex differences in CRF_2_ binding also emerged following puberty [96]. Compared to juvenile males, adult males had increased CRF_2_ binding in the four subnuclei of the amygdala examined (i.e., basolateral, posterodorsal, posteroventral, and central), while CRF_2_ levels remained low following puberty in females. Similarly, in the BNST, which is considered part of the extended amygdala, adult male voles had greater CRF_2_ binding than their female counterparts [97]. Sex differences in CRF receptor binding in the amygdala could contribute to sex differences in anxiety. Blocking or reducing CRF_1_ receptors in the basolateral nucleus of the amygdala decreases anxiety-related behavior [98,99]. If the role of CRF_2_ in the amygdala is to counteract the effects of CRF_1_ activation, like it is in other regions, CRF_2_ activation could decrease CRF_1_-mediated anxiety. Compared to males, female rats have a greater ratio of CRF_1_:CRF_2_ binding in the basolateral nucleus of the amygdala. This sex difference could translate into increased anxiety following stressful events in females, which, if true in humans, would predispose women anxiety disorders such as PTSD.

The fact that sex differences in CRF receptor binding emerge after puberty, implicates the gonadal hormone surges that occur during puberty in these effects [96]. Estrogen, androgen, and progesterone receptors are found in CRF producing regions [49,100-104]. Moreover, androgen treatment increases CRF_2_ mRNA in several brain regions of male rats [105]. In the human myometrium, estrogen and progesterone increase CRF_1_ expression, although this has not yet been demonstrated in the brain [106]. The mechanism underlying gonadal hormone regulation of CRF receptor expression is not fully understood, but putative estrogen, progesterone, and androgen response elements have been identified on the promoter regions of CRF receptor genes [105,107,108]. Thus, direct regulation of gene transcription by gonadal hormones is possible. Together these data suggest that sex difference in CRF_1_ and CRF_2_ binding are likely due to gonadal hormone regulation of CRF receptor number, rather than other mechanisms (e.g., sex differences in differences in receptor affinity for the CRF or neuronal number).

### Sex differences in CRF_1_ receptor signaling

In addition to sex differences in CRF receptor binding, sex differences in CRF_1_ signaling have been identified that can render neurons of females more sensitive to CRF [109-111]. The CRF_1_ is a G protein-coupled receptor that changes conformation upon CRF binding to promote the coupling of GTP binding proteins, which initiate intracellular signaling cascades [51,112]. Although CRF_1_ can promiscuously couple several G proteins, it preferentially binds Gs [112,113]. CRF-induced Gs activation stimulates adenylyl cyclase to form cyclic adenosine monophosphate (cAMP), increasing protein kinase A (PKA) signaling [114,115]. Sex differences in CRF_1_ coupling to Gs have been identified [109]. Specifically, receptor immunoprecipitation of cortical tissue revealed that more Gs was pulled down with the CRF_1_ of females compared to males under unstressed conditions, indicating that the CRF_1_ in female rat cortex is more highly coupled to Gs than in males. Using the same immunoprecipitation technique, it was found that acute stressor exposure (15 min of forced swimming) enhanced CRF_1_-Gs coupling in males to female levels. Stressed females had similarly high levels of CRF_1_-Gs binding as unstressed females. Additionally, these effects were not modulated by circulating ovarian hormones as coupling was comparable in both ovariectomized and intact female rats. The increased binding of CRF_1_ to Gs in females would translate into greater cAMP-PKA signaling in this group. Mice genetically modified to have either greater Gs activation or increased PKA-signaling have an anxiogenic phenotype [116,117]. Thus, the sex difference in CRF_1_-Gs coupling and subsequent signaling could increase anxiety in response to stressful events in females.

Interestingly, sex differences in CRF_1_-Gs coupling may also occur in other brain regions. Although the receptor immunoprecipitation technique is limited to the cortex because of the amount of protein required, the sex difference in CRF_1_-Gs coupling found in cortex mirrors the sex difference in the physiological response of LC neurons to CRF. During a stressful event, CRF released in the LC activates noradrenergic neurons to coordinate arousal, attention, and vigilance [42,118-120]. LC neurons of female rats are more sensitive to CRF than males, such that a dose of CRF that is too low to activate neuronal firing in males increases neuronal firing in females [121]. Acute swim stress enhances LC neuronal sensitivity in males to female levels, but does not alter sensitivity of female neurons [121]. Notably, these sex differences parallel the direction of the sex differences in CRF_1_-Gs coupling in cortex, such that stressed males and females (regardless of stress history) had neurons that were more sensitive to CRF and CRF_1_ receptors that were more highly coupled to Gs than unstressed males. The CRF-induced increase in LC neuronal firing is mediated by the cAMP-PKA signaling cascade [122]. Thus, the sex difference in coupling and subsequent signaling likely causes the sex difference in LC physiology. Consistent with this idea, pretreatment of LC neurons with a cAMP antagonist revealed that the majority of the LC physiological response to CRF in unstressed females and stressed males was cAMP mediated, whereas in unstressed males, only 50% of the LC response to CRF was cAMP-mediated [109]. Together these data suggest that sex differences in CRF_1_-Gs coupling and cAMP-PKA signaling render LC neurons of females more sensitive to CRF. This effect can have consequences for LC-mediated stress responses in females. Typically, activation of the LC-arousal system is thought to be adaptive, as it promotes cognition and behavior aimed at coping with stress [119,123]. However, activation of this system to stress levels by stimuli that should be subthreshold for stress reactivity would be disruptive. Given that female LC neurons are more sensitive to CRF, LC activation is more likely to be inappropriately elicited in females.

### Sex differences in CRF_1_ receptor trafficking

Sex differences in CRF_1_ trafficking also are observed in LC neurons. Like most G-protein coupled receptors, in response to saturating concentrations of ligand, the CRF_1_ receptor is desensitized and internalized (i.e., trafficked from the membrane to the cytosol) [51,124-126]. This is considered a cellular adaptation to high levels of CRF because the internalized receptors can no longer be activated. Both CRF agonist treatment and acute swim stress (15 min) induce CRF_1_ internalization in LC neurons of male rats [109,126,127]. Interestingly, swim stress fails to internalize these receptors in female rats [109]. A failure of CRF_1_ internalization in females could result from sex differences in CRF_1_ binding to β-arrestin, the protein that initiates the internalization process [51,128,129]. Stress increases CRF_1_-β-arrestin binding in cortical tissue in males, but fails to do so in females [109]. If the sex difference in CRF_1_ association with β-arrestin is also present in LC neurons, it would account for sex differences in CRF_1_ trafficking.

**Figure 1:**
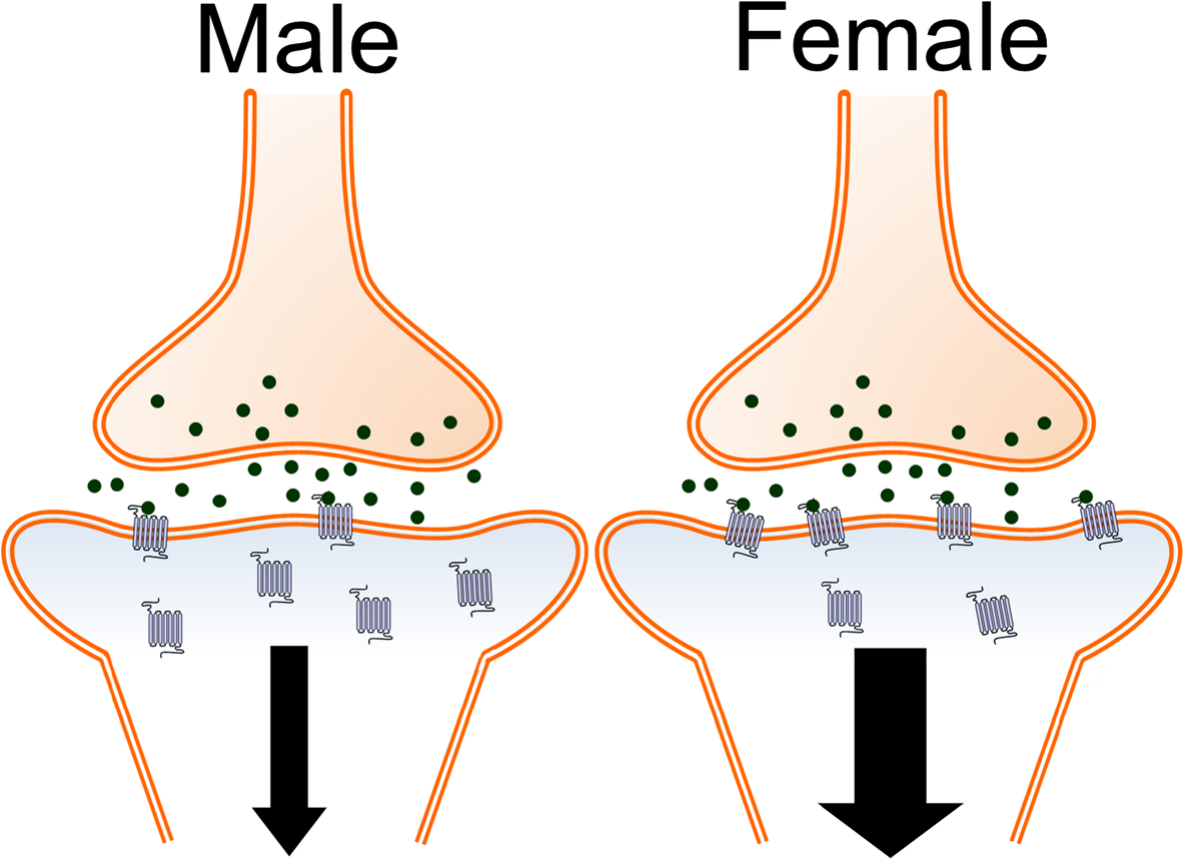
**This schematic illustrates sex differences in CRF_**1 **_trafficking in LC dendrites **[133]**. **Under conditions of excessive release of CRF (green), CRF_1_ receptors (blue) internalize in male neurons (left). This effect reduces neuronal responses to CRF in males. In females (right), CRF_1_ receptors fail to internalize under conditions of CRF hypersecretion, resulting in greater neuronal responses to CRF in this group.

The lack of CRF_1_ internalization observed following stress in females could result in female LC neurons that are more sensitive to conditions of CRF hypersecretion, which as noted, occurs in depression and PTSD. This idea was tested by using CRF overexpressing mice that model conditions of chronic stress and display anxiety-related behavior [130-132]. High levels of CRF expression in the LC region were observed in CRF overexpressing mice, regardless of sex [133]. The firing rates of LC neurons were evaluated in these mice and compared to wild type controls. LC firing rates were similar in male and female wild type mice. However, LC neurons of female CRF overexpressing mice fired roughly 3× faster than male and female wild type controls [133]. Interestingly, the firing rates of male overexpressing mice were maintained at wild type levels. Because CRF expression was high in the LC of male overexpressing mice, this result suggested the presence of some compensatory cellular mechanism in this group. As noted, internalization is a cellular adaptation that can reduce cellular responding to high levels of CRF, so CRF_1_ internalization was evaluated. Much like the trafficking pattern following acute stress in rats, CRF_1_ receptors were internalized in male but not female overexpressing mice [133]. The inability of female CRF_1_ to internalize under conditions of CRF hypersecretion can explain the high LC neuronal firing rate in this group (Figure 1). High levels of LC activation are linked to hyperarousal, a symptom of stress-related disorders that leads to increased agitation, restlessness, re-experiencing, and sleep disturbance [69,134-136]. The aforementioned results suggest that sex differences CRF_1_ modulation of the LC-arousal systems could more easily shift females into this dysregulated state of hyperarousal.

### CRF binding protein (CRF-BP)

In addition to the mechanisms already discussed, the activity of CRF is also regulated by CRF-BP [137-139]. CRF-BP is expressed in the pituitary and in several brain regions including the olfactory bulb, amygdala, BNST, the ventral premammillary and dorsomedial nuclei of the hypothalamus, and sensory relay nuclei in midbrain and brain stem [140]. CRF-BP inhibits CRF-induced ACTH release in pituitary corticotropes *in vitro*, suggesting CRF-BP sequesters free CRF [141]. Centrally, both acute stress and CRF administration increase the expression of CRF-BP in the basolateral nucleus of the amygdala, an effect that may facilitate recovery from stressor exposure [142-144]. However, in the ventral tegmental area, CRF-BP potentiates the signaling of CRF receptors, thereby amplifying the effects of CRF [145]. Interestingly, sex differences in expression of CRF-BP have been observed in the pituitary, with female mice having higher levels of CRF-BP than their male counterparts [146]. Thus, the complex role of CRF-BP in modulating CRF, and its potential to contribute to sex differences in the development of stress-related disorders, certainly warrants further study.

### Glucocorticoids regulate responses to stress

Activation of the HPA axis leads to the release of glucocorticoids. These stress hormones promote physiological changes to adapt to acute stress, such as metabolizing glucose, catabolizing proteins and lipids, and decreasing inflammation [29]. As noted earlier, glucocorticoids also feedback centrally and at the level of the pituitary to terminate the endocrine response to stress. However, if the HPA stress response becomes persistently activated due to impaired negative feedback or other mechanisms, then the adverse state of hypercortisolemia can occur. Hypercortisolemia contributes to the pathophysiology of a number of psychiatric and medical disorders including depression [147-154].

Glucocorticoids act through two receptors: mineralocorticoid receptors (MRs) and glucocorticoid receptors (GRs). MRs are highly expressed in the hippocampus and septum, while GRs are found throughout the brain in regions including the hippocampus, septum, amygdala, hypothalamus, and the nucleus of the solitary tract [155-157]. The affinity of these receptors is different, such that glucocorticoids bind to MRs with higher affinity (K_d_, 1 nM), but to GRs with lower affinity (K_d_, 2.5-5 nM) [155,156,158]. The differential distribution and affinity of these receptors has led to the proposal that MRs maintain the tone of the stress system under normal conditions [155]. In contrast, GRs, because of their lower affinity for the ligand, respond to high levels of glucocorticoids to mediate the physiological recovery from stressful events and restore the system to homeostasis [155,159]. In support of this, there are two phases of glucocorticoid negative feedback, fast and delayed, and both appear to be regulated by GRs. Specifically, fast feedback is thought to be mediated by membrane bound GRs that, when activated, induce endocannabinoid suppression of the hypothalamus [160,161]. Delayed feedback involves the translocation of nuclear GRs from the cytosol to the nucleus where they can repress gene transcription of CRF, CRF_1_, and precursors of ACTH [162-167]. These studies highlight the fact that GRs are the critical receptor subtype that coordinates the recovery of the HPA axis response to stress.

Glucocorticoid negative feedback can be assessed by the dexamethasone suppression test [168-171]. This test was developed to diagnose Cushing’s syndrome, a disorder characterized by excessive endogenous glucocorticoid secretion, but also has been used to assess HPA axis dysregulation in several psychiatric disorders, including depression [168,169,171]. Dexamethasone is a synthetic glucocorticoid that binds with high affinity for GRs [172]. In normal subjects, dexamethasone suppresses the release of cortisol, but it fails to suppress cortisol in 20-50% of depressed patients, indicating impaired negative feedback [169-171]. When the dexamethasone suppression test is combined with the CRF stimulation test, the sensitivity to differentiate between normal and pathological states increases to 80%, suggesting the combined test is a better assessment of the HPA axis dysregulation observed in depression [173-175]. Decreased negative feedback in depression is thought to result in the hypercortisolemia that is often observed in these patients [147-153,176]. Interestingly, there is evidence that HPA axis dysregulation precedes depression, and that cortisol levels and negative feedback normalize with antidepressant treatment or clinical recovery [148,149,151]. Additionally, patients with Cushing’s syndrome often experience depression, and removal of their adrenal glands relieves depressive symptoms [177,178]. Taken together, these finding indicate that changes in glucocorticoid negative feedback may cause depressive symptoms [148,149,151].

### Sex differences in glucocorticoid negative feedback

The most widely reported sex difference in stress response systems is that female rodents have higher levels of HPA axis hormones than males. Specifically, basal levels of glucocorticoids are elevated in female rats relative to their male counterparts [93,179,180]. Stressor exposure induces greater corticosterone release for a longer duration in female than male rodents [91,93,179-185]. In contrast to the consistent findings in rodents, reports of sex differences in cortisol levels in humans are equivocal [186-196]. Despite these discrepancies, mechanisms that can lead to higher levels of glucocorticoids in females have been identified. One such mechanism is greater CRF expression in the hypothalamus of female compared to male rodents, which can translate into greater HPA axis activation [91-94,197]. Another mechanism involves sex differences in glucocorticoid negative feedback. As noted, in response to acute stress, female rodents have a protracted elevation of corticosterone, which is indicative of a slower negative feedback compared to males [91,180,184]. Studies in humans also suggest that feedback is slower in women than men [191-193]. Sex differences in negative feedback may be established by ovarian hormones, because female rats treated with estrogen have a slower glucocorticoid feedback than ovariectomized controls [198,199]. This suggests that estrogen can decrease negative feedback, resulting in a prolonged release of glucocorticoids in females.

Sex differences in glucocorticoid negative feedback may be explained by sex differences in glucocorticoid receptor number. In the pituitary, female rats have fewer MRs and GRs than males [200]. Similarly, compared to male rats, glucocorticoid binding is lower in the hypothalamus of females, likely reflecting fewer receptors [201]. Estrogen regulates the expression of receptors for glucocorticoids, again highlighting the role of estrogen in establishing this sex difference. Specifically, estrogen treatment decreased GR mRNA in breast cancer cells [202]. Also, estrogen treatment of ovariectomized females downregulated GR expression in the hippocampus, hypothalamus, and pituitary [200,203]. Thus, direct downregulation of glucocorticoid receptors by estrogen is possible.

### Sex differences in GR translocation and other feedback regulators

**Figure 2:**
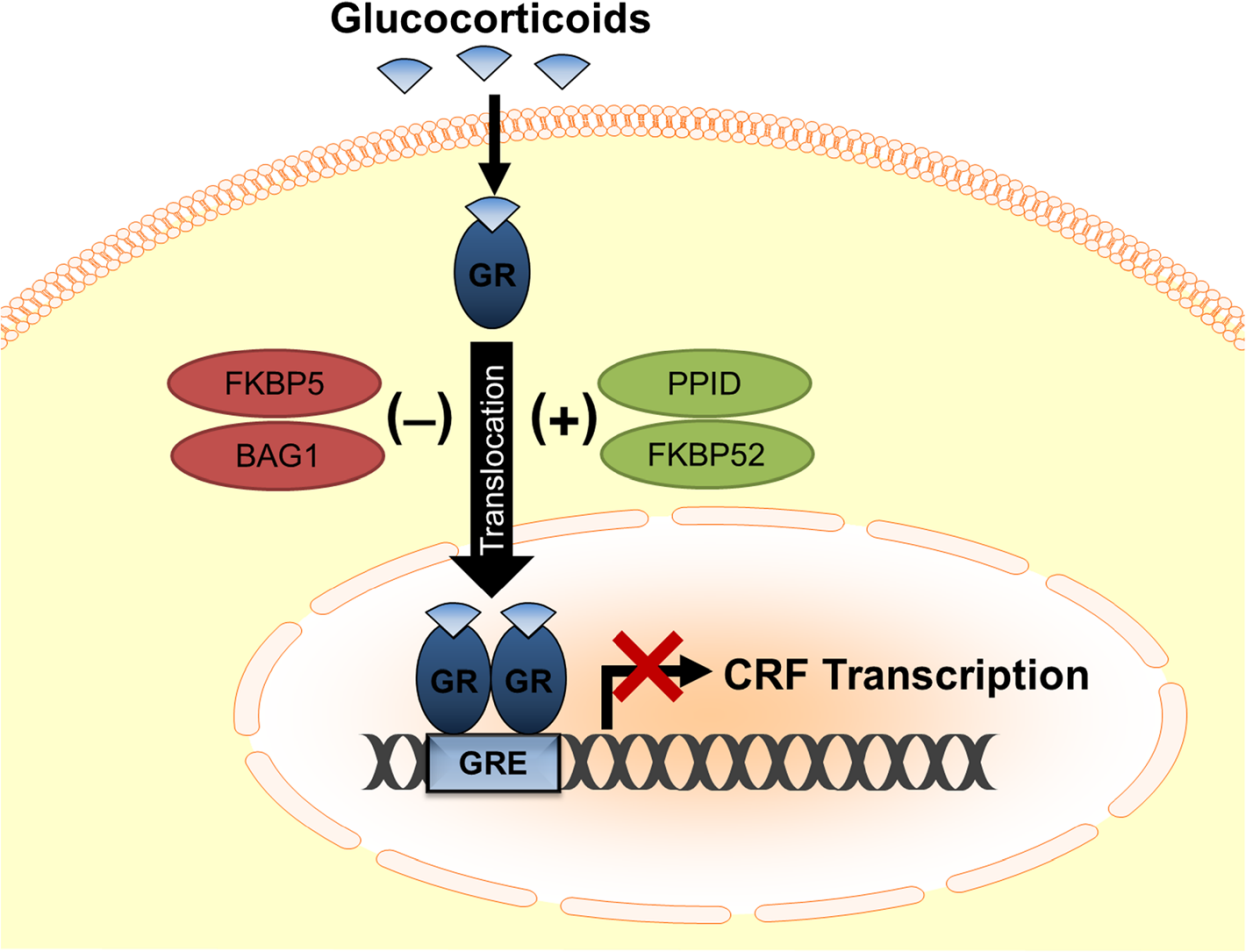
**This schematic depicts the role of GR translocation in glucocorticoid negative feedback. **Glucocorticoids readily cross the plasma membrane to bind nuclear GRs. GRs then are shuttled into the nucleus by multiple co-chaperones (in green), such as PPID and FKBP52. Once inside, GRs bind to glucocorticoid response elements (GREs) on genes to repress transcription of CRF and precursors for ACTH. Other co-chaperones (in red), including Bag1 and Fkbp5, inhibit GR translocation, an effect which can attenuate negative feedback.

Another mechanism by which sex differences in glucocorticoid negative feedback can be established involves GR translocation [204]. The process of translocation is initiated by the binding of glucocorticoids to nuclear GRs. The GRs then are shuttled by multiple co-chaperones (e.g., PPID and FKBP52) into the nucleus to regulate gene transcription [205-207]. In addition to co-chaperones that promote GR translocation, other co-chaperones, such as Bag1 and Fkbp5, inhibit GR translocation, highlighting that this process is highly regulated (Figure 2) [205,207-210]. Bourke and colleagues (2012) identified several co-chaperones that were regulated in a sex-specific manner, and often these sex differences were exacerbated by exposure to chronic stress. Alterations in co-chaperone expression observed in female rats with a history of chronic adolescent stress were linked to changes in glucocorticoid negative feedback [204]. Specifically, when these females were exposed as adults to an acute stress challenge, elevated levels of Bag1 and Fkbp5 were observed. Acute stressor exposure also attenuated GR translocation and negative feedback in this group when compared to females that were not stressed during adolescence. These findings suggest that stress-induced increases in co-chaperones that inhibit GR translocation result in impaired glucocorticoid negative feedback in females. Interestingly, a history of adolescent stress did not alter GR translocation or HPA responses to acute stressor exposure in males, indicating that females are more vulnerable to stress during the adolescent period.

Sex differences in glucocorticoid negative feedback may also occur via mechanisms other than GR expression and translocation. Progesterone can compete with glucocorticoids for binding cites on GR itself, while estrogen receptors can compete with GRs for a binding site on the promoter region of genes, demonstrating that ovarian hormones can directly block glucocorticoid actions [211,212]. Additionally, because estrogen receptors co-localize with GABA neurons in the area around the paraventricular of the hypothalamus nucleus, it has been proposed that estrogen can reduce GABA input into the hypothalamus, thereby indirectly inhibiting negative feedback in females [213]. Together these studies suggest that the regulation of glucocorticoid negative feedback by ovarian hormones is complex and not limited to effects solely at GR regulation. Nevertheless, the reduction in GR levels and the prevention of their translocation into the nucleus in stressed females would decrease the ability of glucocorticoids to repress CRF and ACTH gene transcription. Thus, sex differences at the level of GRs are likely important mechanisms that lead to increased stress hormones levels in women. Under typical conditions, slight increases in glucocorticoid levels due to a protracted negative feedback may not impact women. However, under conditions of chronic stress, the slower termination of the HPA axis response in women could result in high glucocorticoid levels for an extended period of time. As hypercortisolemia is thought to cause depression, sex differences in glucocorticoid negative feedback may predispose women to this disease [148,149,151].

### Implications for pharmacotherapy

The aforementioned studies reveal sex differences at the level of two receptors that mediate responses to stress: CRF_1_ and GR. Not only can these sex differences contribute to the development of mood and anxiety disorders, but they have implications for pharmaceutical treatments. For example, CRF_1_ antagonists are currently being developed to treat stress-related disorders [214-216]. The fact that the CRF_1_ binds Gs and β-arrestin proteins differently in males versus females suggests sex differences in the conformation of this receptor. This conformational change could alter the binding of CRF_1_ antagonists, changing their efficacy in men versus women. Understanding the mechanisms that differentially regulate these receptors in males and females may also lead to novel treatments. Perhaps future pharmacotherapies could be developed to promote β-arrestin binding to CRF_1_, thereby stimulating internalization, a potentially longer lasting treatment than could be achieved with CRF_1_ antagonists. Other compounds targeting co-chaperones to increase GR translocation could accelerate negative feedback of the HPA response. Given the sex differences in CRF_1_internalization and GR translocation, both of these treatments are likely to be particularly effective in women.

These studies also underscore the importance of using female subjects in preclinical research. A review of the non-human animal studies published in 2009 found that single-sex studies of male animals outnumbered those of females 5.5 to 1 in neuroscience and 5 to 1 in pharmacology [217]. Given the evidence that sex differences occur at the receptor level, it is possible that receptor antagonists would work well in one sex but not the other. In fact, one interesting possibility is that the pharmacological treatments discussed above may *only* be effective for the treatment of stress-related disorders in females. Because compounds are typically screened exclusively in male animals, new drugs that appear to be ineffective for males would never get past the preclinical drug development stage, despite the fact that they may be very effective for females. This scenario could deny women with mood and anxiety disorders important treatment options.

## Conclusions

The emerging literature on sex differences in stress-related receptors indicates that, although psychological factors may play a role, biological factors also contribute to sex differences in stress-related psychiatric disorders. Moreover, these studies are not only advancing our understanding of the neurobiological bases for this sex bias, but they also may guide the development of novel pharamotherapies. In addition, the fact that sex differences occur at the receptor level has even broader implications. These are the first reports, to my knowledge, of sex differences in G-protein coupled receptor signaling and steroid hormone receptor translocation. It is possible that similar sex differences occur in other G-protein coupled and steroid hormone receptors. If supported by future studies, sex differences in receptor function may also explain why many other psychiatric disorders (e.g., autism, attention-deficit disorder, etc.) occur at different rates in men and women [218,219]. Thus, sex differences at the receptor level may be an important yet underexplored determinant of vulnerability to a wide range of psychiatric diseases.

## Abbreviations

PTSD: Post-traumatic stress disorder; CRF: Corticotropin releasing factor; GRs: Glucocorticoid receptors; HPA: Hypothalamic pituitary adrenal; CRF_1_: CRF_1_ receptor type; CRF_2_: CRF_2_ receptor type; BNST: Bed nucleus of the stria terminalis; cAMP: cyclic adenosine monophosphate; PKA: Protein kinase A; LC: Locus coeruleus; ACTH: Adrenocorticotropic hormone; MR: Mineralocorticoid receptor; GREs: Glucocorticoid response elements.

## Competing interests

The author has no competing interests.

## Author’s contributions

DAB wrote and edited manuscript, and has read and approved the final version.
